# Supplementary material for: Experimental uninephrectomy associates with less parasympathetic modulation of heart rate and facilitates sodium-dependent arterial hypertension
Source: PLoS One. 2022 Mar 9;17(3):e0265086. doi: 10.1371/journal.pone.0265086 (PMC8906640; doi:10.1371/journal.pone.0265086)
Supplement: Attachment — Submitted filename: Response-to-Reviewers_rb.docx [file pone.0265086.s002.docx]

**Response to Reviewers, manuscript PONE-D-21-32299**

title: „Experimental uninephrectomy associates with less parasympathetic tone and facilitates sodium-dependent arterial hypertension“

PLOS ONE

**Reviewers' comments:**

**Reviewer #1:**

**Pliquett et al. reported that the parasympathetic tone of uninephrectomized mice was decreased, when compared to that of normal mice without uninephrectomy. In addition, the authors indicated that the levels of blood pressure in uninephrectomized mice with salt overload and taurine deficiency were comparable to those in uninephrectomized mice with angiotensin II infusion. This manuscript is important, because the physiological underpinnings of the blood pressure increase after uninephrectomy remain unclear. However, some serious concerns have been raised.**

**(1) It is understandable that, as the authors indicated, it takes a long time to increase blood pressure if structural changes in blood vessels are the main cause. However, even though the rapid change of the decrease of parasympathetic nerve was definitely caused, the levels of blood pressure did not change at all. It is difficult for me to understand this discrepancy of the results. The authors should indicate why the levels of blood pressure by uninephrectomy did not change at all more adequately.**

Answer: In Results section, more results on blood-pressure regulation in uninephrectomized mice were added. In Discussion section, these results were discussed accordingly.

**New** (Page 8, 1st paragraph, last sentence): „However, peak systolic arterial blood pressure tended to be higher in uninephrectomized mice (125.3 ± 20.3 mmHg) as compared to normal controls (114.7 ± 9.5 mmHg), although this difference did not reach the significance level.“

**New** (Page 8, 2nd paragraph, last sentence):

„However, in uninephrectomized mice, a non-significant tendency to a reduced baroreflex sensitivity of heart rate was demonstrated (5.7 ± 3.6 bpm/mmHg versus 6.8 ± 1.6 bpm/mmHg).“

**Old** (Discussion, Page 8, 1st paragraph, 1st sentence): „Here, in a murine model of hypertension using a combination of kidney-mass reduction, salt overload and taurine deficiency, the degree of arterial hypertension was comparable to a high-angiotensin model of hypertension.“

**New** (Discussion, Page 10, 1st paragraph, 1st + 2nd sentence): „Here, in a murine model of kidney-mass reduction, peak systolic arterial blood pressure tended to be higher in comparison to normal controls. In addition, when salt overload and taurine deficiency were applied to uninephrectomized mice, the degree of arterial hypertension was comparable to a high-angiotensin II model of hypertension.“

**Old** (Discussion, Page 8, 1st paragraph, last sentence): „Changes in baroreflex sensitivity were ruled out between uninephrecomized mice and normal controls here, while a model of renovascular hypertension with reduced kidney mass was associated with a reduced baroreflex sensitivity as shown previously in our group (20).“

**New** (Discussion, Page 10, 1st paragraph, last 5 lines): „As for baroreflex sensitivity, in the current study, a tendency to less baroreflex sensitivity was demonstrated in uninephrectomized mice when compared to normal controls. Likewise, in a model of renovascular hypertension with reduced kidney mass, a reduced baroreflex sensitivity was shown previously (19).“

**(2) There is no data to evaluate the difference between uninephrectomized mice with angiotensin II administration or salt load and those without treatment intervention. The results are important. Therefore, the authors should show the results.**

Answer: After sham surgery or after uninephrectomy, we performed 24-hour telemetric recordings 1 week after surgery. Afterwards, in uninephrectomized on salt overload+taurine deficiency or on angiotensin-II treatment, a telemetric 24-hour measurement over 7 consecutive days was performed. Therefore, a direct control group (uninephrectomized mice without intervention) is lacking in this study. However, a comparison of mean and peak systolic arterial pressure at baseline and at the end of study was provided. Methods, Results sections and Figures were revised accordingly.

**New:** (Methods, Page 6, 1st paragraph, last 3 lines): „For analysis, a within-group comparison of daily mean and peak systolic arterial pressure at baseline and at day 7 was performed“

**New** (Methods, Page 7, last paragraph, 3rd-4th line):

„For comparisons within groups, paired Student`s t test was used.“

**Old** (Results, Page 8, 3rd paragraph, first 2 sentences):

„When uninephrectomized mice were exposed to drinking water with added sodium chloride (2%) and L-alanine (2%), the evolution of arterial hypertension was similar to the evolution of arterial hypetension in a group of uninephrecomized mice subjected to a subcutaneous angiotensin-2 infusion.“

**New** (Results, Page 8, 3rd paragraph, first 2 sentences):

„When compared to baseline, uninephrectomized mice subjected to a high-salt and a low-taurine intake had a higher mean systolic arterial pressure (Figure 4) and a higher peak arterial pressure (Figure 5) after one week. Likewise, the positive-control group subjected to angiotensin-II treatment had a higher mean systolic and peak systolic after one week.“

**Old:** Figure 3

**New:** Figure 4 + Figure 5

**(3) The authors showed the data of HF-HR and LF-SBP in page 7, 3rd paragraph. I do not know when those data were measured. The authors should indicate the time more clearly.**

Answer: The times of measurement were specified precisely:

**Old** (Methods, Page 5, 2nd paragraph, 1st + 2nd line): „After a two-week recovery period, a one-hour baseline recording was taken in the morning in unrestrained, resting conditions. The last 30 minutes were used for analysis.“

**New** (Methods, Page 6, 2nd paragraph, first 10 lines): „After one week of recovery from sham surgery or uninephrectomy and during the one-week period of secondary hypertension induction, telemetric blood-pressure readings of individual animals were obtained every 5 minutes until the animals were sacrificed. Pulse intervals (defined as consecutive dP/dt) were extracted from aortic blood pressure waveforms using ART 4.2 Gold software (Data Sciences International; St. Paul, Minnesota, USA). Mean heart rate, mean and peak systolic and diastolic blood pressure were determined over 24 hours. In addition, after recovery following sham surgery or uninephrectomy and 1 week after onset of secondary hypertension, a one-hour continuous baseline recording was taken in the morning in unrestrained, resting conditions. The last 30 minutes were used for powerspectral analysis and for determination of spontaneous baroreflex sensitivity as reported previously (13).“

**(4) The authors indicated that the evolution of arterial hypertension was similar between sodium chloride overload and angiotensin II stimulation in Figure 3. However, the average daily systolic blood pressure was different on 3 of 7 days under investigation. I think that the levels of systolic blood pressure between sodium chloride overload and angiotensin II stimulation are not same. Therefore, the authors should revise the expression adequately.**

Answer: True. The presentation of blood-pressure evolution was revised accordingly.

**Old** (Results, Page 7, 3rd paragraph, first 3 sentences): „When uninephrectomized mice were exposed to drinking water with added sodium chloride (2%) and L-alanine (2%), the evolution of arterial hypertension was similar to the evolution of arterial hypetension in a group of uninephrecomized mice subjected to a subcutaneous angiotensin-2 infusion. Specifically, average daily systolic pressure was not different on 4 of 7 days under investigation. However, the daily maximum systolic blood pressure yielded from day 4 to day 7 was higher in angiotensin-2 - treated animals (Figure 3).“

**New** (Results, Page 8, last paragraph, last 3 lines, Page 9, first paragraph, first 2 lines): „Specifically, during the week of hypertension development, daily mean systolic arterial blood pressure was similar in uninephrecomized mice of both groups on 4 of 7 days under investigation. However, on 5 of 7 days, daily peak systolic blood pressure was higher in the positive control group on angiotensin-II - treatment than in the group on salt-rich, taurin-deficient diet.“

**(5) Angiotensin II is correct and angiotensin 2 is not correct. Therefore, the authors should revise the word adequately.**

Answer: We agree. We corrected this mistake in the manuscript.

**(6) The authors indicated that “As a negative finding, uninephrectomy alone did associate with arterial hypertension in this study.” in page 8, 2nd paragraph. I think that uninephrectomy alone did not associate with arterial hypertension in this study. The authors should revise the sentence adequately.**

Answer: We agree.

**Old** (Discussion, Page 8, 2nd paragraph, 1st sentence): „As a negative finding, uninephrectomy alone did associate with arterial hypertension in this study.“

**New** (Discussion, Page 10, 2nd paragraph, 1st sentence): „As a negative finding, uninephrectomy alone was not associated with arterial hypertension in this study.“

**(7) There are some typographical and grammatical errors in this manuscript. Therefore, the manuscript should be reviewed by the native speaker of English.**

Answer: The revised version of the manuscript was proof-read by a native speaker (Dr. A. Adeabgo).

**Reviewer #2: Pliquett and Brandes present a study examining the effect of uninephrectomy on autonomic tone and the development of hypertension. Spectral analysis of blood pressure and heart rate variability was used as a surrogate for autonomic activity, with the authors reporting that uni-nephrectomy had no differences in resting blood pressure, heart rate or spontaneous baroreflex gain. The LF-HF ratio of SBP was shifted higher, and the HF of PI was lower suggesting a shift towards parasympathetic dominance. Two forms of secondary hypertension were induced – via angiotensin II infusion and a high salt/taurine deficient diet. The time course and magnitude of hypertension development was similar between Ang II and high salt/taurine deficiency.**

**Major Points**

**The overall object of comparing Ang II and high salt/low taurine hypertension is not clear.**

Answer: As objective, we introduced Ang-II-treated mice as a positive control group for mice on a high-salt and low-taurine intake. We revised Methods and Results section accordingly.

**Old** (Methods, Page 4, last paragraph, 2nd sentence): „Two groups of mice of 6 animals each were subjected to uninephrectomy and to angiotensin-2-dependent hypertension or to salt overload and taurin deficiency.“

**New** (Methods, Page 5, last paragraph, lines 4-6): „Afterwards, as an add-on study, uninephrectomized mice were randomly assigned to a model of hypertension due to salt overload and taurine deficiency (n=6) or to angiotensin-II-dependent hypertension model as a positive control group (n=6).“

**Old** (Results, Page 8, 3rd paragraph, subtitle and first 2 sentences):

„**Uninephrectomized mice show similar propensity of hypertension following sodium-chloride overload and following angiotensin-2 stimulation.**

When uninephrectomized mice were exposed to drinking water with added sodium chloride (2%) and L-alanine (2%), the evolution of arterial hypertension was similar to the evolution of arterial hypetension in a group of uninephrecomized mice subjected to a subcutaneous angiotensin-2 infusion.“

**New** (Results, Page 8, 3rd paragraph, subtitle and first 2 sentences):

„**Uninephrectomized mice with sodium-chloride overload and taurine deficiency show a predisposition for hypertension**

When compared to baseline, uninephrectomized mice subjected to a high-salt and a low-taurine intake had a higher mean systolic arterial pressure (Figure 4) and a higher peak arterial pressure (Figure 5) after one week. Likewise, the positive-control group subjected to angiotensin-II treatment had a higher mean systolic and peak systolic after one week.“

**The Introduction pitches the heightened cardiovascular risk faced by kidney donors, or those born with one kidney. Is the intension to evaluate the impact of uninephrectomy on the propensity to hypertension when faced with the “challenge” of the two hypertensive models? If so, the main statistical comparisons should be between sham nephrectomy and uninephrectomy, with (1) Ang II and (2) high salt/low taurine.**

Answer: It is the intention to point at the sequelae of uninephrectomy in terms of a predisposition for hypertension. As a specific challenge, a high-salt-low-taurine intake was introduced. Ang-II treatment served as a positive control group. Both Introduction and Methods were modified, to convey this study goal.

**Old** (Introduction, Page 3, 2nd paragraph, 5th sentence): „In the present study, the influence of both uninephrectomy, sodium-chloride overload and taurin deficiency on autonomic nervous system function and on blood-pressure regulation was investigated.“

**New** (Introduction, Page 3, 3rd paragraph, lines 1-4): „In the present study, the influence of uninephrectomy both on blood-pressure and autonomic-nervous-system regulation was investigated. In addition to uninephrectomy, the role of a hypertensive challenge on autonomic nervous system function and on blood-pressure regulation was investigated. As a hypertensive challenge, a high-salt-low-taurine diet was applied.“

**Old** (Methods, Page 4, last paragraph, 2nd sentence): „Two groups of mice of 6 animals each were subjected to uninephrectomy and to angiotensin-2-dependent hypertension or to salt overload and taurin deficiency.“

**New** (Methods, Page 5, 2nd paragraph, lines 4-6): „Afterwards, as an add-on study, uninephrectomized mice were randomly assigned to a model of hypertension due to salt overload and taurine deficiency (n=6) or to angiotensin-II-dependent hypertension model as a positive control group (n=6).“

**It isn’t clear how comparing the rise in BP between these two quite different experimental models of hypertension gives any insight into the impact of uninephrectomy itself; surely the magnitude of hypertension with Ang II is simply dependant on the dosage. Also, given that the clinical prevalence of low taurine is extremely rare, the real-world relevance of this model needs a clear justification in the text.**

Answer: The impact of uninephrectomy was assessed by hemodynamic data in comparison to sham surgery and by assessing the blood-pressure response to high-salt, low-taurine intake. In the revised manuscript, a comparison of daily mean and peak systolic arterial pressure at baseline versus at end of intervention was performed (revised Figure 4 and Figure 5). The positive control group applying Ang-II treatment in uninephrectomized mice illustrates the magnitude of the hypertensive effect of the intervention (high-salt, low-taurine intake). As for the intervention, a low taurine intake by virtue of high L-alanine intake allows for less taurine-associated attenuation of hypertension. The antihypertensive effect of taurine was demonstrated by Sun and colleagues (reference 12).

**The manuscript contains many grammatical, tense and wording errors. Given that PLOS-One does not copy edit, the authors should consider engaging a proof-reader to improve the readability.**

The revised version of the manuscript was read by a native speaker (Dr. A. Adeabgo).

**Minor Points**

**The abstract states that blood pressure measurements were made “non-invasively” – the surgical implantation of the telemetry catheter is an invasive procedure. I think what the authors mean is that telemetry recordings were made from conscious, undisturbed animals in their home cages. The term “non-invasive blood pressure” in rodents usually refers to plethysmography.**

True. „non-invasively“ is not adequate. Now, this part was revised.

**Old** (Abstract, Page 2, 2nd paragraph, lines 4-5): „Non-invasive telemetry readings of heart-rate and blood-pressure were obtained.“

**New** (Abstract, Page 2, 2nd paragraph, lines 4-5): „A transfemoral aortic catheter with telemetry unit was implanted, telemetry readings of heart-rate and blood-pressure were recorded.“

**The opening paragraph of the introduction raises some valid concerns around uninephrectomy but is rather vague – it would be helpful to give some numbers around the increased risk for hypertension in the patient groups mentioned.**

Answer: We added specific information.

**New** (Introduction, Page 3, 1st paragraph, lines 4-6): „Specifically, hypertension developed in 4%, 10%, and 51% of kidney donors at 5, 10, and 40 years, respectively (2), and the development of hypertension among kidney donors prevailed after correction for aging (3).”

**The numbers in each group should be clearly stated near the beginning of the Methods.**

Answer: Patient numbers (per group) were added.

**Given that telemetric recording of BP is a very well-established and commonly used technique, the authors might consider reducing the detail around implantation and give a reference instead?**

Answer: True. In the revised manuscript, a reference is provided

**Old** (Methods, Page 4, 1st paragraph, last 3 sentences): „Telemetry catheters were implanted in all animals as described previously (13). In short, the left femoral artery was chosen for surgical implantation of the aortic catheter. The attached telemetry unit TA11PAC10 (Data Sciences International, St. Paul, Minnesota, USA) was placed subcutaneously on the right flank.“

**New** (Methods, Page 5, 2nd paragraph, last 3 lines): „Transfemoral aortic catheters with telemetry units (TA11PAC10, Data Sciences International, St. Paul, Minnesota, USA) were implanted in all animals as described previously (13).“

**The authors refer to Mean HR, BP etc being determined over 24h, but elsewhere indicate that recordings were made for 60 minutes each day.**

Answer: In the revised manuscript, the respective sections were revised:

**Old** (Methods, Page 5, 2nd paragraph, lines 1-2): „After a two-week recovery period, a one-hour baseline recording was taken in the morning in unrestrained, resting conditions. The last 30 minutes were used for analysis.“

**New** (Methods, Page 6, 2nd paragraph, lines 1-10): „After one week of recovery from sham surgery or uninephrectomy and during the one-week period of secondary hypertension induction, telemetric blood-pressure readings of individual animals were obtained every 5 minutes until the animals were sacrificed. Pulse intervals (defined as consecutive dP/dt) were extracted from aortic blood pressure waveforms using ART 4.2 Gold software (Data Sciences International; St. Paul, Minnesota, USA). Mean heart rate, mean and peak systolic and diastolic blood pressure were determined over 24 hours. In addition, after recovery following sham surgery or uninephrectomy and 1 week after onset of secondary hypertension, a one-hour continuous baseline recording was taken in the morning in unrestrained, resting conditions. The last 30 minutes were used for powerspectral analysis and for determination of spontaneous baroreflex sensitivity as reported previously (13).“

**It is more common to express HRV in terms of the LF:HF ratio of PI, rather than HR alone. This is because it is possible for both LF and HF power to change. It is less common to report the LF:HF ratio for SBP as representing “autonomic balance”.**

Answer: LF-SBP:HF-HR ratio is regarded as a measure of sympathovagal balance in mice, as LF:HF ratio derived from powerspectrum of heart rate represents sympatho-vagal balance in humans. In mice, the corresponding frequency bands for sympathetic and vagal tone were published by V. Baudrie and colleagues (Reference 15). We adhered to this methodology. We are aware that other groups determined powerspectral LF- and HF bands in mice, e.g. Chiara Piantoni and colleagues published in Front Neurosci. 2021;15:617698. However, in this publication, only total intensity and parasympathetic intensity of the HF band were reported. The intensities of the LF band of frequency domain were not reported. As an explanation, the LF band does not convey information on sympathetic tone in mice.

**The authors mention “baroreflex curves” – this is not correct for spontaneous baroreflex gain technique they have used; the full curve can generally only be examined using the Oxford technique, whereas sBRG examines ramps in BP (ie generally only on the linear portion of the baroreflex curve).**

Answer: True. We corrected this mistake.

**Old** (Methods, Page 5 (last 4 lines), Page 6 (first 5 lines): „Baroreflex sensitivity of heart rate was determined by the sequence technique of concomitant changes of systolic blood pressure and pulse intervals (digitized, linearly interpolated) utilizing the Hemolab software (17-19). Concomitant changes of systolic blood pressure (of at least 15 mmHg) and pulse intervals of at least 4 consecutive heart beats were correlated. For individual baroreflex curves, a correlation coefficient of at least 0.9 was mandated for analysis. In addition, a time delay of 0 seconds was chosen for analysis of concomitant blood-pressure and pulse-interval changes. The average of at least 10 individual baroreflex slopes (linear portion of systolic blood pressure – pulse-interval relationship) was considered as baroreflex sensitivity of heart rate.

**New** (Methods, Page 7, 1st paragraph, last 9 lines): „Baroreflex sensitivity of heart rate was determined by spontaneous baroreflex gain technique. Sequences of concomitant spontaneous changes of systolic blood pressure (of at least 15 mmHg) and pulse intervals of at least 4 consecutive heart beats were digitized, linearly interpolated and correlated utilizing the Hemolab software (17;18). For individual ramps in blood pressure and pulse intervals, a correlation coefficient of at least 0.9 was set as threshold for data inclusion for the analysis. A time delay of 0 seconds was chosen for analysis of concomitant blood-pressure and pulse-interval changes. The average of at least 10 individual baroreflex ramps (linear portion of systolic blood pressure – pulse-interval relationship) was considered as baroreflex sensitivity of heart rate.“

**In paragraph 2 of the discussion, the authors state that “uninephrectomy alone did associate with arterial hypertension” – assume this should be “did not”?**

True, this point was corrected.

**Old** (Discussion, Page 8, 2nd paragraph, 1st sentence): „As a negative finding, uninephrectomy alone did associate with arterial hypertension in this study.“

**New** (Discussion, Page 10, 2nd paragraph, 1st sentence): „As a negative finding, uninephrectomy alone was not associated with arterial hypertension in this study.“

**Figures 1 and 2 could be combined, and Figure 3 consider maybe showing just MAP rather than SBP/DBP in separate graphs, and perhaps add in HR?**

In merged Figure 2, a graph on heart rate was added, a graph on LF-SBP:HF-HR ratio was omitted.

**The data availability statement does not appear compatible with PLOS-One guidelines, as no reason for restricting access to “request from authors” is given.**

Answer: The data availability statement was revised. An ethical issue was named for restricted data access.

**Reviewer #3: The purpose of this study was to investigate the effect of unilaterial nephrectomy on autonomic tone and arterial blood pressure in mice. Normotensive C57Bl6N mice were subjected to sham surgery or unilateral nephrectomy. Mice were also instrumented with telemetric blood pressure sensors to monitor arterial blood pressure and heart rate. Apparently the nephrectomized mice were treated with subcutaneous infusions of angiotensin II or a sodium chloride-enriched, taurine-deficient diet. The exact protocol is not clear from the Methods section and therefore, the results are difficult to understand and to interpret. Based on spectral analysis of heart rate and blood pressure, the authors conclude that unilateral nephrectomy is associated with reduced parasympathetic tone and that the combination of unilateral nephrectomy and taurine-deficiency predisposes to sodium-chloride sensitive hypertension.**

**General Comments**

**The study addresses a potentially important topic. While it is known that patients with only one kidney (e.g., living donor) have an elevated risk for developing hypertension, the exact mechanism for this elevated risk is unknown. The experimental approach with telemetric blood pressure recordings and drug application using osmotic minipumps is technically challenging.**

**Major Comments**

**1. The manuscript lacks a specific description of the experimental protocol and results. For example, the exact time course of the protocol is not outlined. It is unclear at which time point after the nephrectomy the data were collected. I think a Figure showing the timeline of the experimental protocol would be helpful. Apparently, the telemetric blood pressure sensors were implanted first and one week later mice were subjected to a second surgery, where the nephrectomy or sham procedure was performed. It is unclear, at which time the osmotic minipumps were implanted and if pumps were replaced after 14 days (it is stated that the mice were observed for 4-6 weeks).**

Answer: Osmotic minipumps were not changed as stated on Page 4, last paragraph, 6th line („The osmotic minipumps were used to a maximum duration of administration of 14 days.“). The respective statement that mice were observed for 4-6 weeks was revised.

**Old** (Methods, Page 5, 1st and 2nd line): „After observing the mice for 4 - 6 weeks, the mice were sacrificed under isoflurane anaesthesia by decapitation.“

**New** (Methods, Page 6, 1st paragraph, lines 5-6): „After finishing the experiments, the mice were sacrificed by decapitation under isoflurane anaesthesia.“

**2. Apparently, there were 3 groups (but not 100% sure from the Methods section): (1) A control group without nephrectomy but sham surgery that was not treated with angiotensin or high-salt diet; (2) a unilaterally nephrectomized group with angiotensin II infusion (for how long?); and (3) a unilaterally nephrectomized group with high-salt-low taurine diet. However, Figs. 1 and 2 only show two groups. Is the nephrectomized group the one with angiotension II or with high-salt-low taurine diet? Also Fig. 1 shows no effect on blood pressure, but Fig. 3 shows an increase in blood pressure in the nephrectomized groups. I believe that I don’t understand the protocol and at which time the different data were obtained. I strongly feel that the experimental protocol needs to be described in much more detail.**

Answer: Figure 1 showing the sequence of interventions was added. There were 2 groups: (1) normal controls without nephrectomy, but sham surgery, (2) uninephrectomized mice. In an add-on experiment, uninephrectomized mice were randomly assigned to a high-salt-low-taurine diet or to an angiotensin-II treatment. Method section was revised accordingly.

**Old** (Methods, Page 4, lines 11-19): „Telemetry catheters were implanted in all animals as described previously (13). In short, the left femoral artery was chosen for surgical implantation of the aortic catheter. The attached telemetry unit TA11PAC10 (Data Sciences International, St. Paul, Minnesota, USA) was placed subcutaneously on the right flank.

One week later, one group of mice was subjected to a sham surgery (skin incision plus manipulation on one kidney). Two groups of mice of 6 animals each were subjected to uninephrectomy and to angiotensin-2-dependent hypertension or to salt overload and taurin deficiency.“

**New** (Methods, Page 5, 1st paragraph, lines 8-9 and lines 12-14, 2^nd^ paragraph, lines 1-6):

„The experimental setting was displayed in Figure 1. ... Transfemoral aortic catheters with telemetry units (TA11PAC10, Data Sciences International, St. Paul, Minnesota, USA) were implanted in all animals as described previously (13).

One week later, one group of mice was subjected to a sham surgery (n=10) which consisted of skin incision plus manipulation of one kidney. Another group was subjected to uninephrectomy (n=15). After one week of recovery, hemodynamic parameters were obtained and powerspectral analysis performed as outlined below. Afterwards, as an add-on study, uninephrectomized mice were randomly assigned to a model of hypertension due to salt overload and taurine deficiency (n=6) or to angiotensin-II-dependent hypertension model as a positive control group (n=6).“

**3. The experimental procedures are technically challenging in mice and mice may not recover easily from these type of interventions (telemetric sensor, osmotic minipumps are relatively large in respect to the small size of the animals). I wonder if more reliable data could have been obtained if the study would have been conducted in larger animals, such as rats. I strongly feel the authors need to explain why mice were used for this study.**

True. Physiological studies on kidney-mass reduction need to be performed in larger rodents and other animal models. Mice were chosen to be able to perform add-on studies on genetically modified animals with and without kidney-mass reduction.

**4. The Introduction needs a clearly stated hypothesis and rationale why the study was conducted.**

Answer: We agree.

**New** (Introduction, Page 3: last 2 lines, Page 4, lines 1-2): „Here we hypothesized that, firstly, blood pressure is higher in uninephrectomized mice than in normal controls. Secondly, uninephrectomy correlates with a predisposition for hypertension after a high-salt-low-taurine intake and also with changes in sympathetic and/or parasympathetic tone, when compared with normal controls.“

**5. Page 5, towards the bottom: LF-SBP is not a measure of sympathetic tone. It is a measure of sympathetic modulation of vascular tone. For example, if sympathetic nerve activity is high but there is reduced vascular sympathetic responsiveness, there would be low LF-SBP but high sympathetic tone. Likewise, HF-HR is not a measure of parasympathetic tone. It is a measure of parasympathetic modulation of sinus node function. Again, parasympathetic tone may be high but if the sinus node has reduced parasympathetic responsiveness, there would be reduced HF-HR despite high parasympathetic tone. I suggest changing the wording throughout the manuscript accordingly.**

Answer: We agree. Changes were implemented accordingly.

**6. Typically, sympathovagal balance is calculated as the ratio of LF-HR to HF-HR. The low frequency component of heart rate variability (not systolic blood pressure variability) is used. The ratio of LF-SBP to HF-HR is comparing sympathetic effects on vascular tone with parasympathetic effects on the sinus node. I don’t think this is a valid marker of sympathovagal balance. Sympathovagal balance should consider the same target organ (i.e., the sinus node).**

Answer: Both frequency domains (LF-SBP, HF-HR) of powerspectral analysis correlated with sympathetic modulation and parasympathetic modulation in mice (Baudrie and colleagues (Reference 15)). Therefore, we introduce the ratio LF-SBP:HF-HR as a surrogate of the classic LF:HF ratio, i.e. sympathovagal balance, in humans. LF band of the frequency domain does not convey information on sympathetic modulation in mice.

**7. It is unclear where the data from Figs. 1 and 2 come from. One column is labeled as “Uninephrectomized”. However, my understanding of the protocol is that there were two “uninephrectomized” groups. Which one is shown in Figs. 1 and 2 and why is the other uninephrectomized group not shown? You may include the data from the sham operated group in Fig. 3. All figure legends should list the number of animals for each data point shown.**

Answer: The new Figure 1 and the revised Methods section clearly describe the 2 groups displayed in the merged Figure 2 (formerly Figure 1 and 2). We gratefully accept this point.

**8. What is causing the decrease in parasympathetic modulation of sinus node function in the mice with unilateral nephrectomy? If the authors propose a role for afferent renal nerve traffic, renal denervation experiments should be performed to test this hypothesis.**

Answer: Discussion section was expanded accordingly.

**New** (Discussion, Page 11, last 4 lines): „The present study provides a rationale for experiments on renal salt handling in uninephrectomized mice with or without renal denervation in order to reduce afferent renal autonomic nerve traffic, which, hypothetically, is involved in salt-sensitive hypertension there.“

**Minor Comments**

**1. Abstract, Results section, 1st line: …were not different “n” uninephrectomized mice … “n” should be “in”.** Corrected.

**2. Keywords: Avoid keywords that are already in the title.** Keywords were revised accordingly.

**3. Page 7, line 6 from bottom: “hypetension” should read “hypertension”.** Corrected.

**4. Page 7, bottom, last line: the numbers need units.** Corrected.

**5. Page 9, 1st line: … a dedicated study … (not studies).** Corrected.

**6. Page 8, line 10 from bottom, “model” not “models”.** Corrected.

**7. References: Some of the references are dated. Is there newer literature on some of these topics?**

Answer: One reference was removed (Ref. 18), one reference (Ref. 24) was replaced by a more relevant one (Tahara A et al.). Discussion was revised accordingly:

**Old** (Page 8, 3rd paragraph, 1st sentence): „As for salt overload, the adaptative processes following uninephrectomy including proximal-tubular hypertrophy (23) may translate into a higher sodium reabsorption (24).“

**New** (Page 10, 2nd paragraph, lines 5-9): „As for salt overload, the adaptative processes following uninephrectomy include proximal-tubular hypertrophy (22). A beneficial role of sodium-glucose-cotransporter-2 (SGLT-2) inhibition was shown in uninephrectomized KK/Ay type 2 diabetic mice providing indirect evidence that SGLT-2 is upregulated when kidney mass is reduced (23).“
