# Supplementary material for: Experimental uninephrectomy associates with less parasympathetic modulation of heart rate and facilitates sodium-dependent arterial hypertension
Source: PLoS One. 2022 Mar 9;17(3):e0265086. doi: 10.1371/journal.pone.0265086 (PMC8906640; doi:10.1371/journal.pone.0265086)
Supplement: Attachment — Submitted filename: Response to Reviewers.docx [file pone.0265086.s003.docx]

Reviewer #2: Thank you to the authors for the considerable revisions to this paper, which have greatly improved clarity and readability. In particular, the scientific writing is much improved, which makes reviewing the manuscript markedly easier. There remain some significant issues with the experimental design and interpretation of results.

We thank the reviewer for reevaluating our manuscript. Below, please find our point-by-point reply.

The statement “uninephrectomy and taurine-deficiency predisposes to salt-sensitive hypertension” is not supported by the data. To demonstrate that uninephrectomy makes a subject more vulnerable to hypertension, the authors would need to show that taurine-deficiency and high salt do not induce hypertension in a sham group (or that the magnitude of hypertension was less, or the onset delayed in sham animals).

We agree with this statement. A sham group was not used in this study. The conclusions (Abstract), the Introduction and the Discussion have been changed accordingly:

*Old (Page 2, Abstract, Conclusions):*

„The combination of uninephrectomy and taurine-deficiency predisposes to sodium-chloride sensitive hypertension.“

*New (Page 2, Abstract, Conclusions):*

„The combination of uninephrectomy, taurine-deficiency and high-salt intake led to arterial hypertension.“

*Old (Introduction, Page 3 (last 2 lines) and 4 (first 2 lines)):*

„Here we hypothesized that, firstly, blood pressure is higher in uninephrectomized mice than in normal controls. Secondly, uninephrectomy correlates with a predisposition for hypertension after a high-salt-low-taurine intake and also with changes in sympathetic and/or parasympathetic tone, when compared with normal controls.“

*New (Introduction, Page 3 (last 2 lines) and 4 (first 2 lines)):*

„Here we hypothesized, firstly, that blood pressure is higher in uninephrectomized mice than in normal controls, and secondly, that uninephrectomy leads to changes in sympathetic tone and/or in parasympathetic modulation of sinus node function when compared with normal controls. In addition, we hypothesized that salt overload with taurine deficiency increases systolic blood pressure in uninephrectomized mice to a comparable extent as in hypertensive mice on a standard dose of subcutaneous angiotensin-2 stimulation.“

*Old (Discussion, Page 11, 2nd + 3rd paragraph):*

„However, to the best of our knowledge, salt overload alone did not increase blood pressure in an animal model. Rather, a sodium- chloride overload was shown to predispose to angiotensin-II mediated hypertension (29).

In summary, in uninephrectomized mice, the compensatory increase of sodium-chloride reabsorption by the remaining kidney may predispose to arterial hypertension in the context of an increased oral salt load and taurine deficiency. The reduced vagal modulation of heart rate may represent another underlying pathomechanism for hypertension following uninephrectomy. The present study provides a rationale for experiments on renal salt handling in uninephrectomized mice with or without renal denervation in order to reduce afferent renal autonomic nerve traffic, which, hypothetically, is involved in salt-sensitive hypertension there.“

*New (Discussion, Page 11, 2nd + 3rd paragraph):*

„However, salt overload alone did not increase blood pressure in an animal model. Rather, a salt overload was shown to predispose to angiotensin-II mediated hypertension (29).

In summary, in the context of uninephrectomy with increased oral salt load and taurine deficiency, arterial hypertension was present. In uninephrectomized animals without increased salt load and taurine deficiency, a reduced vagal modulation of heart rate was found. Future studies conducted over longer observation periods need to determine, whether autonomic changes following uninephrectomy represent a pathomechanism for hypertension. In addition, the present study provides a rationale for experiments on renal salt handling in uninephrectomized mice with or without renal denervation in order to reduce afferent renal autonomic nerve traffic, which, hypothetically, is involved in salt-sensitive hypertension.“

There remains no clear reason to include the Ang II “positive control” group. The authors state that “during the week of hypertension development…mean BP was similar (between Ang II and taurine/NaCl) on 4/7 days, but peak BP was higher in 5/7 days”. This is not a meaningful comparison, as the rate of onset and the degree of hypertension with Ang II is almost entirely dependant on the dose given – repeating this study with a lower or higher dose of Ang II selected would almost certainly give different findings. If the comparison were instead between an identical dose of Ang II in uninephrectomized vs sham groups, then the authors could draw some valid conclusions about the susceptibility to Ang II hypertension.

We agree that the design of these study groups does not allow for a comparison as far as susceptibility to Ang II is concerned. Therefore, any judgement on the degree of hypertension reached by high-salt, taurine-deficient diet was avoided. The term „similar“ was removed from the abstract, the Discussion was modified:

*Old (Page 9, Discussion, lines 2-6):*

„In addition, when salt overload and taurine deficiency were applied to uninephrectomized mice, the degree of arterial hypertension was comparable to a high-angiotensin II model of hypertension. While the high – angiotensin II model was associated with sympathoactivation, the parasympathetic modulation of heart rate was decreased in uninephrectomized mice, as compared to normal mice without a reduced kidney mass.“

*New (Page 9, Discussion, lines 2-6):*

„While the high – angiotensin II model associated with sympathoactivation, uninephrectomized mice showed less parasympathetic modulation of sinus node function as compared to normal controls without a reduced kidney mass. In addition, when salt overload and taurine deficiency were applied to uninephrectomized mice, arterial hypertension developed within the same time frame as in the angiotensin-II model of hypertension.“

In the revised manuscript, we described the evolution of hypertension in uninephrectomized mice on a high-salt and taurine-deficient diet in comparison to the established Ang II model of hypertension using a standard dose of Ang II. Specifically, we used the dose of Ang II (1.4 mg per kg body weight), as published previously:…I believe that the authors have misinterpreted how to apply the literature on the spectral analysis of blood pressure and heart rate in mice and invite them to carefully reconsider (and potentially re-analyze) their results. The papers cited (Baudrie, 2007 with an editorial commentary by Stauss, 2007) are investigations of the appropriate frequency bands specific to mice, including validation using various drugs to block components of the autonomic nervous system. The authors are correct that the literature most clearly supports the LF(SBP) power (0.15-0.6 Hz) generally correlating with sympathetic vasomotor tone, and the HF(PI) power (2.5-5 Hz) with cardiac vagal tone (largely respiratory). However, it is definitely not appropriate to combine the power outputs from SBP and PI into a single LF:HF ratio. I cannot find any examples of this in the literature, in any species. If the authors wish, they may like to report the LF:HF ratios for both SBP and PI separately. But in general, the evidence is more supportive of the LF:HF ratio for PI being the most appropriate indication of cardiac autonomic balance – the LF:HF ratio for SBP is rarely reported (unlike LF(SBP) alone). Thireau et al have published a guide on performing spectral analysis in mice (https://physoc.onlinelibrary.wiley.com/doi/10.1113/expphysiol.2007.040733) or the authors could refer to many more recent publications where frequency domain analyses of HR and BP in mice have been published

Thank you for this comment on the methodology used to determine surrogates of sympathetic tone and on parasympathetic modulation of heart rate. Now, we omitted the presentation of sympathetic-parasympathetic ratio (LF-SBP/HR-HF). The protocol by Thireau et al. stated (under the Heading „Short-term ECG analysis“ between Figure 4 and Figure 5) that “LF and HF were significantly diminished by atropine (LF, 2.48 ± 0.23 *versus* 0.89 ± 0.12 ms^2^; and HF, 2.82 ± 0.34 *versus* 0.94 ± 0.11 ms^2^, *P* < 0.01, before and after injection, respectively).“

Therefore, in mice, the cumulative intensity of the LF-band of the powerspectrum of heart rate was not used as a surrogate of sympathetic modulation of heart rate. In fact, in mice, the LF-band of heart rate is regarded as a means to gauge parasympathetic modulation of heart rate, e.g. by Dr. Volkmar Gross (Max-Delbrück-Centre, Berlin, Germany) for a LF band between 0.25–1.0 Hz): Costa-Goncalves AC, Tank J, Plehm R, Diedrich A, Todiras M, Gollasch M, Heuser A, Wellner M, Bader M, Jordan J, Luft FC, Gross V. Role of the multidomain protein spinophilin in blood pressure and cardiac function regulation. Hypertension. 2008; 52: 702–707. There, the authors state (under the Heading „Autonomic Imbalance in db/db Mice“): „Low-frequency systolic BP (LF-SBP) oscillations are produced by sympathetic modulation of vascular tone. ...In mice, low-frequency HR oscillations are mediated through parasympathetic mechanisms.19 In contrast, in human subjects, low-frequency HR oscillations result from sympathetic and parasympathetic activation.“

Obst M, Tank J, Plehm R, Blumer KJ, Diedrich A, Jordan J, Luft FC, Gross V. NO-dependent blood pressure regulation in RGS2-deficient mice. Am J Physiol Regul Integr Comp Physiol. 2006 Apr;290(4):R1012-9. doi: 10.1152/ajpregu.00288.2005. Epub 2005 Nov 3. There, the authors reflect the topic of parasympathetic control of the LF-band in Figure 5.

Likewise, a different group (Véronique Baudrie et al. Am J Physiol Regul Integr Comp Physiol 292: R904–R912, 2007) reported, that „Atropine also significantly reduced the LF component of the PI spectrum.“

In the current study, we used the HF band (between 2.5 and 5 Hz) as a measure of parasympathetic modulation of heart reate in accordance with the guidance provided by Thireau et al. (<https://physoc.onlinelibrary.wiley.com/doi/10.1113/expphysiol.2007.040733>)

With respect to the following references: <https://www.ncbi.nlm.nih.gov/pmc/articles/PMC5989555/>, <https://www.nature.com/articles/s41598-017-17690-7>, <https://www.frontiersin.org/articles/10.3389/fnins.2021.617698/full> There, an ECG was obtained allowing for R-wave detection. As we used pulse intervals calculated by intervals between consecutive blood pressure peaks (first derivative of peak systolic blood pressure or dp/dtmax) the following reference applies to the revised manuscript: <https://physoc.onlinelibrary.wiley.com/doi/full/10.14814/phy2.12811>)

There, the methodology for sympathovagal balance of the heart cited the following reference:

Montano, N., T. G. Ruscone, A. Porta, F. Lombardi, M. Pagani, and A. Malliani. 1994. Power spectrum analysis of heart rate variability to assess the changes in sympathovagal balance during graded orthostatic tilt. Circulation 90:1826–1831. This reference (a study performed in human volunteers) is not applicable to mice for the reasons mentioned above (LF-band of heart rate does not represent sympathetic modulation of heart rate in mice).

Minor

Introduction “hypertension developed…in kidney donors at 5, 10 and 40 years” should add “years post-transplant” (assuming this is what is meant?) to avoid confusion with the preceding sentence about children born with 1 kidney developing hypertension by the age of 18.

Thank you, we changed this sentence accordingly.

The methods now read “telemetric blood pressure readings…were obtained every 5 minutes until animals were sacrificed”. Could the authors simply state how long each scheduled recording of BP was for? Ie 30s every 5 mins

We agree and implemented this change.

*Old (Page 6, 2nd paragraph, first sentence):*

“After one week of recovery from sham surgery or uninephrectomy and during the one-week period of secondary hypertension induction, telemetric blood-pressure readings of individual animals were obtained every 5 minutes for until the animals were sacrificed.“

*New (Page 6, 2nd paragraph, first sentence):*

“After one week of recovery from sham surgery or uninephrectomy and during the one-week period of secondary hypertension induction, telemetric blood-pressure readings of individual animals were obtained every 5 minutes for 10 seconds until the animals were sacrificed.”

Reviewer #3: The revised manuscript has been improved. Specifically, adding the new Fig. 1 helps to better understand the protocol. I still have a couple of issues that should be addressed:

(comments and page numbers refer to the red-lined version)

1. Abstract, Methods section: “A transfemoral aortic catheter with telemetric unit was implanted “and” telemetry readings …”

Thank you. The 2nd mention of „telemetry“ was deleted.

2. Abstract, Results and Conclusions: These sections still refer to “parasympathetic tone”, which was not really assessed in this study. Please use the term “parasympathetic modulation of sinus node function” instead.

This correction was made throughout the text of the revised manuscript.

3. Page 4, Methods, subsection “Animal Models”: I think Darmstadt is in Hessen (not Hesse, although the local dialect in Darmstadt may call it “Hesse”).

The name of the federal state was changed accordingly, thanks.

4. Throughout the manuscript: present vs. past tense does not appear to be used properly.

We revised the manuscript accordingly.

5. Page 6 top: How can the pulse interval be defined as “consecutive dP/dt”? Maybe dP/dtmax was used to identify the start and end of individual heart beats?

Correct, the manuscript was changed accordingly.

6. Page 6 bottom: “quantitative measure of sympathetic modulation” should read “… of sympathetic modulation of vascular tone”. Likewise “parasympathetic modulation of sinus node function” (not just modulation).

Thank you for this point, we changed the text accordingly.

7. Again, I disagree with the use of the LF-SBP to HF-HR ratio. This ratio does not make any sense physiologically. You are building a ration of responses in different target organs. Furthermore, this ratio is not really needed for the purpose of this study. Since there was no indication of any effects on sympathetic modulation of vascular tone, the use of a ratio is not needed. You can simply use the measured of parasympathetic modulation of sinus node function. With this regard, I think you should calculate and report RMSSD to supplement the frequency-domain analysis and confirm the HF spectral power data.

Thank you, the term sympathovagal balance and the respective data were omitted. Time-domain analysis including HRV, RMSSD was not performed in this study due to the relatively short continuous recordings (over 1 hour) for data analysis. Except for those continuous data measurements for analysis for frequency domain data, the telemetric blood pressure readings were obtained every 5 minutes for 10 seconds. To perform time-domain analysis in mice, continuous heart-rate data would be needed over 24 hours (Thireau et al, https://physoc.onlinelibrary.wiley.com/doi/10.1113/expphysiol.2007.040733).

8. Figure 2: The values on the y-axis for LF-SBP are wrong. A spectral power of 80,000 mmHg2 would correspond to an amplitude of blood pressure fluctuations of 280 mmHg !!! (not possible). Please check your numbers. I think you forgot to multiply with the spectral resolution (but I am not sure). Anyway, these data are wrong.

The calculation of LF-SBP was performed as outlined in Methods. We used Dataquest ART 4.2 Gold software and adhered to the Methodology provided by the manufacturer (Data Sciences International). Data analysis was performed in the same way as reported previously (Reference 13 in the manuscript). The difference in the range of the data (Y axis) to data reported elsewhere may be due to differences in interpolation of systolic blood-pressure data performed prior to Fourier transformation when using different software packages.

9. Throughout the Results section the references to Figure numbers do not match with the actual Figures.

The corrections were made accordingly, thank you.

10. The first paragraph of the Discussion is redundant and simply repeats the Results. This paragraph should be deleted and replaced with a meaningful discussion of the data.

The first paragraph was re-written, redundancies were removed.

11. Summary paragraph at the end of the Discussion: Capital T for the sentence starting in “The present study …”. Last word of this paragraph “… hypertension there”. I don’t think you need the word “there”.

Change were made as pointed out, thanks.

12. If uninephrectomy really were associated with less cardiac parasympathetic tone, why then was heart rate not higher?

With exception of a tendency to (higher) maximum blood pressure, hemodynamic data including heart rate were not different at the end of this short-term study.

Now, nominal heart rate has been reported in the Results section to point at a slight decrease of the minimal and maximal heart rate in uninephrectomized mice under resting conditions likely due to baroreflex activation to buffer a small increase of blood pressure.

*New (Page 8, Results, first paragraph, last sentence):*

„Likewise, although mean heart rate was not different (uninephrectomized mice: 460.7 bpm, normal controls: 462.4 bpm), uninephrectomized mice reached a lower miminal (336.5 bpm) and maximal (563.9 bpm) heart rate than controls (minimal heart rate:403.4 bpm, maximal heart rate: 567.6 bpm) under resting conditions.“

*Old (Page 10, Discussion, second paragraph, first and second sentence):*

As a negative finding, uninephrectomy alone was not associated with arterial hypertension in this study. This may relate to the relatively short observation time of 1 week.

*New (Page 10, Discussion, second paragraph, first sentence):*

“As a negative finding, uninephrectomy alone was not associated with arterial hypertension in this study, even though the nominal reduction of resting minimal and maximal heart rate is consistent with an activation of baroreflex to buffer a small rise of blood pressure during the relatively short observation time of 1 week.”
