# Supplementary material for: Experimental uninephrectomy associates with less parasympathetic modulation of heart rate and facilitates sodium-dependent arterial hypertension
Source: PLoS One. 2022 Mar 9;17(3):e0265086. doi: 10.1371/journal.pone.0265086 (PMC8906640; doi:10.1371/journal.pone.0265086)
Supplement: Attachment — Submitted filename: Response to Reviewers-R3.docx [file pone.0265086.s004.docx]

Reviewer #3:

We thank the reviewer for reevaluating our manuscript. Below, please find our reply.

Except my previous comment #8, the authors have addressed all my previous comments appropriately. However, the data for LF BPV shown in Figure 2 are wrong and not compatible with life. The animals would likely be dead if BPV was that high. A LF spectral power of BP of 80,000 mmHg2 is simply not possible !!!! The fact that the authors reported incorrect LF spectral powers in Fig. 4 of their previous publication (Ref 13), does not make these data correct. Again, my assumption is that the authors forgot to multiply the spectral powers with the spectral resolution, which was 0.019 Hz in this study. If the spectral powers reported in Fig. 2 of the current manuscript are multiplied with 0.019 Hz, the values would make sense. I.e., 80,000 mmHg2 (corresponding to BP amplitudes of 282 mmHg!) would become 1,520 mmHg2 (corresponding to BP amplitudes of 39 mmHg, which may be possible in hypertensive animals).

We thank you for this important comment. You are right, after reviewing the data analysis again, the multiplication of spectral powers with the spectral resolution is lacking. Now, we multiplied all spectral powers with the spectral resolution (0.019 Hz) both in this study and in the previous study cited as reference 13. There, we submit an *erratum*.

Figure 2 was changed, source data was updated.

Results (Page 9, last sentence) was updated:

Old: “As for the autonomic nervous system, parasympathetic modulation of heart rate (HF-HR) was not different between both hypertensive groups, however, sympathetic modulation (LF-SBP) was higher in the high-angiotensin-II model (105988 ± 6739 mmHg² versus 84424 ± 4604 mmHg², p=0.0038).”

New: “As for the autonomic nervous system, parasympathetic modulation of heart rate (HF-HR) was not different between both hypertensive groups, however, sympathetic modulation of vascular tone (LF-SBP) was higher in the high-angiotensin-II model (2014 ± 128.0 mmHg² versus 1604 ± 87.5 mmHg², p=0.0038).”
